# Macrophages as potential targets in gene therapy for cancer treatment

**DOI:** 10.37349/etat.2023.00124

**Published:** 2023-02-28

**Authors:** Yuanzheng Huang, Zhihui Wang, Junni Gong, Dandan Zhu, Wang Chen, Fangzhou Li, Xing-Jie Liang, Xiaoxuan Liu

**Affiliations:** 1 State Key Laboratory of Natural Medicines and Jiangsu Key Laboratory of Drug Discovery for Metabolic Diseases, Center of Advanced Pharmaceuticals and Biomaterials, China Pharmaceutical University, Nanjing 210009, Jiangsu, China; 2 Chinese Academy of Sciences (CAS) Key Laboratory for Biomedical Effects of Nanomaterials and Nanosafety, CAS Center for Excellence in Nanoscience, National Center for Nanoscience and Technology of China, Beijing 100190, China; 3 Nano Science and Technology Institute, University of Chinese Academy of Sciences, Beijing 100049, China; Donghua University, China

**Keywords:** Macrophages, gene therapy, cancer

## Abstract

Macrophages, as ubiquitous and functionally diverse immune cells, play a central role in innate immunity and initiate adaptive immunity. Especially, tumor-associated macrophages (TAMs) are crucial contributors to the tumorigenesis and development of cancer. Thus, macrophages are emerging potential targets for cancer treatment. Among the numerous targeted therapeutic options, gene therapy is one of the most potential therapeutic strategies via directly and specifically regulating biological functions of macrophages at the gene level for cancer treatment. This short review briefly introduces the characteristics of macrophage populations, the functions of TAM in the occurrence, and the progress of cancer. It also summarized some representative examples to highlight the current progress in TAM-targeted gene therapy. The review hopes to provide new insights into macrophage-targeted gene therapy for precision cancer therapy.

## Introduction

Macrophages are the most plastic cells of the hematopoietic system, with a ubiquitous presence in the body and considerable functional diversity. They eliminate pathogens and dead cells through phagocytosis, induce inflammatory responses, mediate immune responses, as well as promote tissue repair and regeneration, and then regulate host homeostasis [1, 2]. This plasticity of macrophages can be attributed to their distinct transcriptional profiles in response to changes in the tissue environment, which in turn triggers adaptive morphologic and functional changes in the cells [3, 4]. Moreover, the homeostatic functions of macrophages can be disrupted by long-term chronic injuries, such as chronic inflammation and tumor progression [5]. In response to persistent infections or chronic irritation, macrophages could mainly differentiate into pro-inflammatory phenotypes and secrete pro-inflammatory cytokines, such as tumor necrosis factor-α (TNF-α) and interleukin 6 (IL-6), to sustain the chronic inflammation which is thought to be closely related to the initiation and promotion of tumors [6]. Once tumors become established, tumor-associated macrophages (TAMs) in the tumor microenvironment (TME) will differentiate from pro-inflammatory phenotype M1 macrophages to trophic immunosuppressive phenotype M2 macrophages, thus promoting the creation of tumor immunosuppressive microenvironments and exacerbating the progression and malignancy of the tumor [7]. Therefore, targeting TAMs is emerging as a novel and attractive therapeutic approach for cancer treatment [8].

Among the current TAM-targeted therapeutic strategies, gene therapy could directly and specifically modulate specific pathogenic genes via nucleic acid therapeutics, which is one of the most potential therapeutic options to regulate the function of macrophages [9]. The review first introduced the origin, classification, differentiation types, and functions of macrophages, and their roles in the occurrence and development of tumors. And then briefly presented the prospects and challenges of TAM-targeted gene therapy for cancer treatment. Moreover, some representative therapeutic targets in TAM and their nucleic acid therapeutics have been summarized. Then some typical examples of TAM-targeted delivery of those nucleic acid therapeutics have been provided to highlight the current progress of TAM-targeted gene therapy for precision cancer medicine.

## Origin, classification, and distribution of macrophages

The discovery of macrophages can be traced back to the second half of the 19th century when the theory of humoral immunity believed soluble factors present in the serum and bodily secretions (later identified as antibodies), and not cells, were exclusively responsible for immunity [10]. This dogma was challenged in 1883 when Metschnikoff observed migratory cells engulfing foreign substances, and named the cells ‘phagocytes’ (from the Greek ‘phago’ meaning to devour, and ‘cytos’ meaning cell) and the process ‘phagocytosis’ [10]. Metschnikoff [11] defined phagocytes as cells that can discriminate self from non-self, recognize and engulf cell debris, senescent cells, and invading pathogens, and kill engulfed bacteria through enzymes (cytases). In 1887, Metschnikoff further classified phagocytes as “macrophages” and “microphages” (now called neutrophils) on the basis of their functions [12]. Since then, more and more cells that possessed the typical characteristics mentioned above were identified as macrophages.

**Figure 1.**
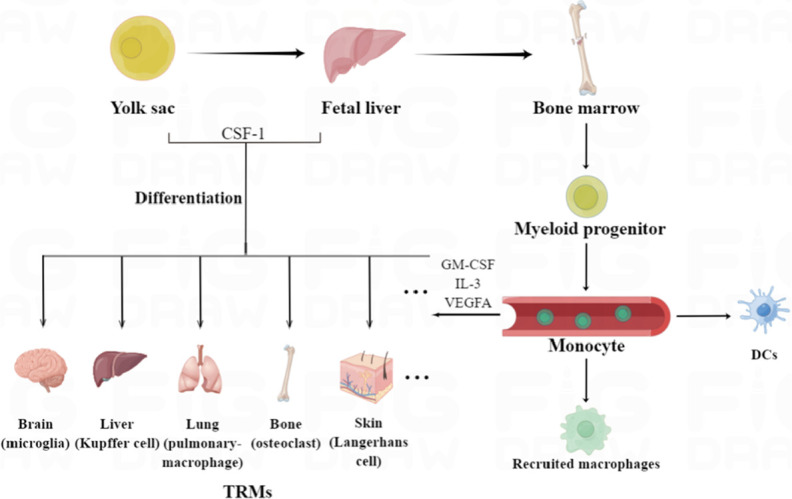
Origins, classification, and distribution of macrophages. Macrophages originate from yolk sac progenitors, fetal liver, and bone marrow, and can be divided into TRMs and recruited macrophages. TRMs mainly originate from the yolk sac or fetal liver or both, and can also be differentiated from monocytes in blood circulation. The recruited macrophages were mainly derived from myeloid monocytes. GM-CSF: granulocyte-macrophage-CSF; VEGFA: vascular endothelial growth factor A. DCs: dendritic cells. This figure was drawn by Figdraw (www.figdraw.com)

In general, macrophages are believed to originate from yolk sac progenitors, fetal liver, and bone marrow, and each of these distinct lineages persists into adulthood (Figure 1) [13]. They are not only present in the blood, but also widely distributed in tissues and organs. The macrophages present in tissues are collectively called tissue-resident macrophages (TRMs) [14], such as the macrophages in connective tissues, lymph nodes, spleen, and pleural cavity, Kupffer cells in the liver, dust cells in the lung, microglia in the brain and osteoclasts in bone tissues. Most TRMs were thought to originate from the yolk sac or fetal liver or both. For example, lineage-tracing experiments have shown that microglia are mainly derived from yolk sac progenitor cells [15], while Langerhans cells originate from the fetal liver and yolk sac [16]. And some TRMs can also be differentiated from bone marrow-derived monocytes [17]. Furthermore, Soucie et al. [18] found that TRMs can self-renew via transient downregulation of the endogenous transcription factors v-maf avian musculoaponeurotic fibrosarcoma oncogene homolog B (MafB) and c-musculoaponeurotic fibrosarcoma oncogene homolog (c-Maf), which are normally dormant [19]. In addition to TRMs, the monocyte-derived macrophages were called “recruited” macrophages, and their differentiations are induced by distinct cytokines and chemokines [20]. For example, the macrophage colony-stimulating factor 1 (CSF-1) receptor (CSF-1R) is a master lineage regulator of nearly all macrophages [21], and its targeted ablation leads to a significant reduction in the macrophages in multiple tissues [22]. The differentiation and self-renewal of macrophages are affected by microenvironmental signals, which underscore their plasticity and diversity in normal physiological processes as well as disease development.

**Figure 2.**
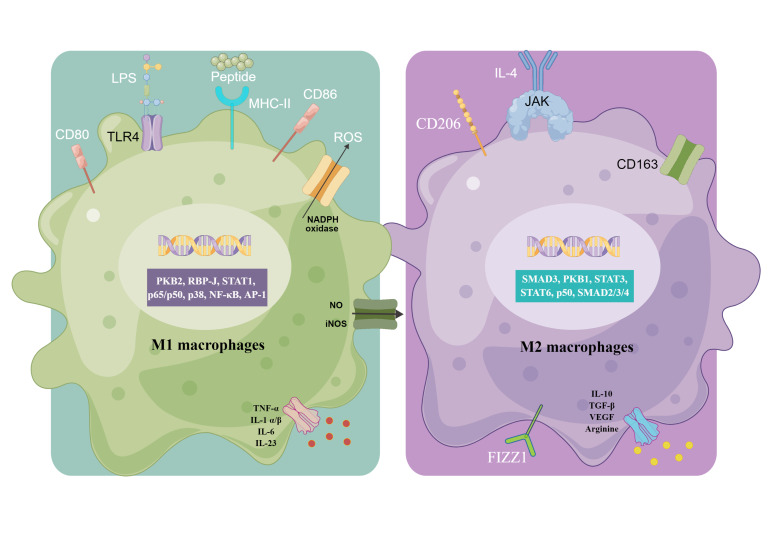
Macrophage polarization and specific functions of M1 and M2 macrophages. Different stimuli activate the generation of M1 and M2 macrophages. ROS: reactive oxygen species; NADPH: nicotinamide adenine dinucleotide phosphate; NO: nitric oxide; iNOS: inducible NO synthase. This figure was drawn by Figdraw (www.figdraw.com)

Macrophages can respond to environmental signals, such as microbial products, foreign substances, damaged cells, and activated lymphocytes, and differentiate into distinct functional phenotypes. Currently, the macrophages are classified into the classically activated M1 and alternately activated M2 phenotypes (Figure 2) [23, 24], which were first identified by Mackaness [25] and Mills [26] respectively. Macrophages are polarized to the M1 phenotype by activated T-helper 1 (Th1) lymphocytes, interferon γ (IFN-γ), pathogenic antigens such as lipopolysaccharides (LPS), and TNF-α through toll-like receptors (TLRs), major histocompatibility complex II (MHC-II), CD80 and CD86. The activated M1 macrophages release pro-inflammatory factors including TNF-α, IL-1α/β, IL-6, IL-23, etc., along with high levels of reactive nitrogen and oxygen intermediates that have strong bactericidal and tumoricidal effects [27, 28]. While the M2 macrophages are alternatively activated by Th2 lymphocytes, IL-4, IL-13, IL-10, and glucocorticoids, they will express high levels of scavenger receptors (e.g., CD163), mannose receptor (e.g., CD206), lectin-receptors (e.g., CD209) and resistin [e.g., found in inflammatory zone 1 (FIZZ1)]. The M2 macrophages can release anti-inflammatory and pro-repair factors such as IL-10, transforming growth factor-β (TGF-β), arginine, and VEGF. Given their immunoregulatory functions, the M2 macrophages are largely involved in parasite containment, tissue remodeling, and tumor progression [29, 30]. The polarization of macrophages is regulated by multiple signaling pathways including Janus kinase (JAK)/signal transducer and activator of transcription (STAT), phosphoinositide 3-kinase (PI3K)/protein kinase B (PKB), Jun N-terminal kinase (JNK), Notch, etc., and then causes changes in related genes. PKB2, recombination signal binding protein J (RBP-J), STAT1, protein 65 (p65)/p50, p38, nuclear factor-kappaB (NF-κB) and activator protein-1 (AP-1) activate genes involved in the M1 phenotype, and the M2-specific transcription factors include small mothers against decapentaplegic 3 (SMAD3), PKB1, STAT3, STAT6, p50/p50 and SMAD2/3/4 [27].

Nevertheless, macrophages exist across a spectrum of activation states more than M1 and M2 types due to multiple stimulatory and inhibitory factors that often act simultaneously. Therefore, the binary classification method is being refined as more phenotypes that are discovered [31]. A recent study also showed that there is considerable phenotypic heterogeneity among macrophages that exist in the same environment, and are derived from the same monocyte pool [32, 33]. These intermediate phenotypes represent the complex physiological functions of macrophages in different microenvironments [34, 35]. Moreover, the M1-M2 phenotypes are reversible to some extent, which reflects the dynamic nature and functions of the macrophages [36, 37].

## Role of macrophages in tumor progression

**Figure 3.**
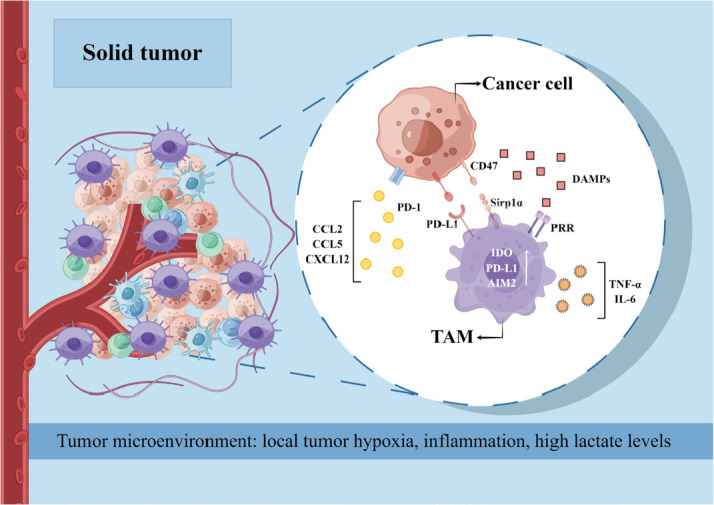
The role of TAMs in tumor progression and related mechanisms. PD-1: programmed cell death protein 1; PD-L1: PD-ligand 1; PRR: (pro)renin receptor; IDO: indoleamine-2,3-dioxygenase; AIM2: absent in melanoma 2; Sirp1α: signal regulatory protein 1 alpha. This figure was drawn by Figdraw (www.figdraw.com)

Uncontrolled inflammatory immune response and tumor immunosuppressive microenvironment are considered to be the dominant driving force in cancer development [38], which still represent an immense obstacle to cancer therapy. TAMs are the most abundant immune cell population within solid tumors and account for approximately 50% of the hematopoietic cells in the TME [39]. Although macrophages have long been regarded as the primary mediator of the anti-tumor immune response, studies increasingly suggest that TAMs promote tumor initiation, progression, and metastasis (Figure 3) [40]. The TAMs are derived from both circulating monocytes as well as TRMs [41]. Sustained low-level stimulation by tumor-derived growth factors, cytokines, and damage-associated molecular patterns (DAMPs) drives myeloid differentiation into monocytes and myeloid-derived suppressor cells (MDSCs). The monocytes are then recruited by chemokines [such as C-C chemokine ligand types 2 (CCL2), CCL5, and C-X-C chemokine ligand 12 (CXCL12)] produced by early cancer cells into the TME, wherein they differentiate into macrophages [42]. Furthermore, the TRMs are activated by the soluble factors produced by cancer cells and other changes in the TME and assist in monocyte recruitment and their differentiation into TAMs [43]. The abundance of TRMs during tumor development depends on the type of cancer, e.g., TRMs in breast tumors decrease over time, while the TRM population in the pancreatic tissues expands as the tumor grows [44].

Following tumor initiation, the majority of the TAMs are derived from monocyte differentiation. Although the exact mechanisms are unknown, local tumor hypoxia, inflammation, and high lactate levels are conducive to the differentiation of recruited monocytes [45]. The macrophages rapidly adapt to the unique TME stimuli, which are responsible for the considerable diversity of TAMs seen across different cancer types and even within the same tumors [46]. The phenotypic pattern of TAMs is overall similar to that of M2 macrophages. For example, the TME shares several characteristics of damaged tissues and wounds, such as the infiltration of inflammatory cells like neutrophils, monocytes, and macrophages, tissue remodeling, and enhanced coagulation [47]. However, tissue injury and healing in the TME are not sequential but instead simultaneous and persistent, which can be attributed to the co-existence of M1 and M2 macrophages. While the M1 macrophages produce TNF-α, IL-6, and chemokines that maintain the inflammatory environment in the tumor, the M2 macrophages promote angiogenesis, growth, and metastasis of tumor tissue [48]. Furthermore, Azizi et al. [49] found that M1 and M2-associated genes are frequently co-expressed in the same tumor cells and correlate positively with one another along the same activation trajectory. These results challenge the binary macrophage polarization model wherein the M1 and M2 activation states are mutually exclusive. This phenomenon has been observed in various tumors [50].

Surprisingly, the pathways that promote cancer-associated inflammation and expansion of immunosuppressive TAMs are virtually the same eliciting protective pro-inflammatory immune responses against pathogens but do not culminate in anti-tumor immune responses [51]. This is due to that activation of pro-inflammatory pathways in TAMs may increase the expression of inhibitory receptors and ligands as well, thereby favoring an immunosuppressive milieu [52]. For example, TAMs express T cell immunoglobulin domain and mucin domain 3 (Tim-3), Tim-4, PD-1, and PD-L1, which can inhibit phagocytosis, inflammasome activation, and production of effector cytokines. In addition, once the macrophages engulf cancer cells, the internalized tumor DNA activates AIM2, which in turn cleaves cyclic guanosine monophosphate-adenosine monophosphate (cyclic GMP-AMP) synthase (cGAS) and upregulates the immunosuppressive PD-L1 and recombinant IDO. Therefore, PD-L1 blockade can enhance the anti-tumor immune response by restoring macrophage activation and proliferation [53].

Thus, the aberrant activation and function of macrophages frequently accompany the disease process. TAMs are critical players in tumor growth, migration, invasion, metastasis, angiogenesis, immunosuppression, and resistance to radiotherapy and chemotherapy [54, 55], making them attractive therapeutic targets for cancer therapy. Currently, three main macrophage-targeting strategies are employed for cancer treatment: i) inhibition of monocyte/macrophage recruitment [56]; ii) killing or depletion of macrophages [57]; iii) reprogramming of macrophage phenotypes at disease sites [58]. Given the plasticity and complexity of macrophages, the conventional treatment approaches often fail to effectively re-establish macrophage homeostasis. Recent advances in functional genomics and gene therapy offer potential strategies to regulate and remodel macrophages for cancer treatment.

## Gene therapy targeting TAMs for cancer treatment

TAM-targeted gene therapy is a promising therapeutic approach for cancer treatment by introducing exogenous nucleic acids to specifically regulate or correct disease-causing/associating genes in TAMs. However, nucleic acid molecules have low stability and poor bioavailability as they can be easily degraded by nucleases and have relatively high molecular weight with intense negative charges, hence difficult to cross cell membranes. Nucleic acids also have a short half-life and are vulnerable to clearance by the liver and kidney [59]. Moreover, macrophages are the primary phagocytes with an abundant enzyme to exacerbate the degradation of nucleic acids. Given the widespread distribution and diversity of macrophages, it is also crucial to minimize mononuclear phygocyte system (MPS) clearance and maximize recognition of specific macrophages to improve targeting efficiency [60]. Therefore, targeted delivery of nucleic acid therapeutics in TAM by safe and efficient vectors is quite essential for regulating the functions of TAMs to achieve successful TAM-targeted gene therapy.

**Figure 4.**
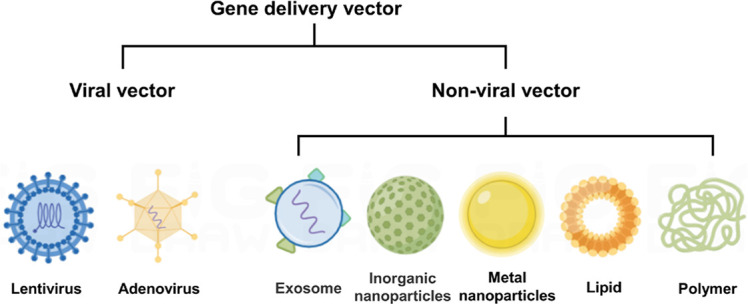
A common type of delivery vehicles for nucleic acid therapeutics. This figure was drawn by Figdraw (www.figdraw.com)

Various materials have been employed as delivery vehicles for nucleic acid therapeutics (Figure 4). Among them, lipid and polymer vectors, are the most advanced and intensively studied nucleic acid delivery vectors, due to their relative simplicity, versatility, and good safety [61, 62]. They could effectively condense nucleic acids to form stable complexes via electrostatic interaction, protect the cargo from enzymatic degradation, and facilitate efficient delivery of nucleic acids into targeted cells for their therapeutic effect. Moreover, the delivery vehicles can be functionalized with different ligands, including small molecules [63], peptides [64], proteins [65], and aptamers [66], to achieve macrophage targeting [67]. For example, mannose and folic acid (FA) recognize CD206 and FA receptors that are highly expressed on macrophages, respectively [68–70]; macrophage-binding peptide [cysteine-arginine-valine (CRV), sequence CRVLRSGSC, where the terminal cysteines form a disulfide bond to render the peptide cyclic] specifically targets the TAMs by recognizing the retinoid X receptor beta (RXRB), which is expressed at significantly higher levels in TAMs compared to other macrophages [71]. Therefore, the delivery vectors with the aforementioned functionalization could improve the recognition of macrophage and achieve efficient uptake of nucleic acid therapeutics in TAM.

**Figure 5.**
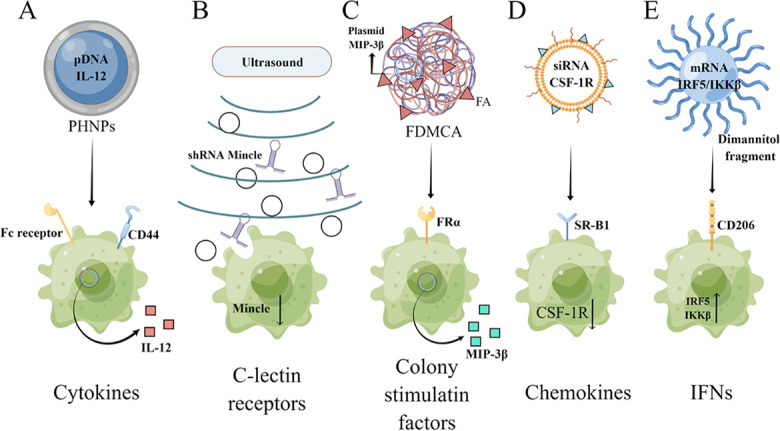
The therapeutic targets in TAMs and the corresponding nucleic acid therapeutics. PHNPs: porous hollow nanoparticles; Fc: crystallizable fragment; Mincle: macrophage-inducible C-type lectin; MIP-3β: macrophage inflammatory response protein 3β; FRα: folate receptor alpha; SR-B1: scavenger receptor B type 1; IRF5: interferon regulatory factor 5; IKKβ: inhibitor of kappa B kinase; FDMCA: FA-modified poly(ethylene glycol)-polycaprolactone-methoxy poly(ethylene glycol) (MPEG)-polylactic acid. This figure was drawn by Figdraw (www.figdraw.com)

With the advances in genetics and bioinformatics technologies, increasing therapeutic targets in TAM have been identified, and different types of nucleic acid therapeutics, including plasmid DNA (pDNA), messenger RNA (mRNA), small interfering RNA (siRNA), small hairpin RNA (shRNA), microRNA (miRNA) and so on [72], have been developed to regulate the function of TAMs, e.g., altering their phenotype or inhibiting the expression of specific receptors, such as cytokines, C-lectin receptors, CSFs, chemokines, and IFNs among others (Figure 5). This review will then show some representative examples of these nucleic acid therapeutics and their delivery strategies.

Cytokines are soluble signaling proteins that can rapidly initiate the response of the immune system to external stimuli. They can regulate immune proliferation, differentiation, and effector function, and are crucial for immune cells to fight against tumor cells [73]. IL-12, a heterodimeric cytokine, can promote the repolarization of macrophages from the M2 to M1 phenotype and thus enhance anti-tumor immune responses [74]. To this end, He et al. [75] developed polypeptide/hyaluronic acid (HA)/protamine/calcium carbonate (CaCO_3_)/DNA nanoparticles (PHNPs) to deliver *IL-12* gene-encoding pDNA (pDNA *IL-12*) into TAMs and cancer cells. The nanoparticles were modified with fusion peptides containing tuftsin sequences that can bind to Fc receptors and neuropilin-1, and HA that can interact with CD44. The PHNPs effectively targeted the macrophages and were able to repolarize the macrophages and reverse tumor immunosuppression.

C-type lectins (CTLs) are a superfamily of proteins and play an important role in innate and adaptive antimicrobial immune responses and the development of immune diseases [76]. The C-type lectin domain family 4 member E [(Clec4e), also known as Mincle] is a pattern recognition receptor that is expressed on macrophages. It was found to be closely related to the production of the trophic immunosuppressive phenotype of TAMs [77]. Tumor cells undergoing necroptosis release the spliceosome-associated protein 130 (SAP130) protein, which binds to Mincle and accelerates tumor growth [78]. In addition, a novel Mincle/spleen tyrosine kinase (Syk)/NF-κB signaling pathway was identified in two syngeneic mouse models with murine melanoma B16F10 cells and lung carcinoma Lewis lung carcinoma cells.7 (LLC.7) xenografts and found to maintain the pro-tumor activities of TAMs by suppressing the M1 phenotype [79]. Thus, Mincle is a key receptor in TAMs and a potential target for anti-tumor immunotherapy. However, no specific Mincle inhibitor has been developed so far, which hinders its clinical translation. A virus-free strategy was recently devised to target Mincle by combining RNA interference with an ultrasound-microbubble-mediated gene transfer system (USMB) [80]. Briefly, an shRNA sequence specific for Mincle was designed, which silenced the expression of Mincle in A549 and A375 xenografts *in vitro* and *in vivo*, and inhibited tumor growth without apparent side effects.

Chemokines are small secreted proteins involved in immune cell trafficking and lymphoid tissue development. They are the largest subfamily of cytokines and can be further subdivided into the cysteine-cysteine (CC), cysteine-X-cysteine (CXC), X-cysteine (XC) and cysteine-X3-cysteine (CX3C) types [81]. MIP-3β is a chemokine of the CC family and mediates chemotaxis and maturation of DCs, and the activation of T cells and other immune cells. Adachi et al. [82] designed a chimeric antigen receptor (CAR)-T cell that can produce MIP-3β and chemotactically attract T cells and DCs to the tumor tissues and thus improving the clearance of tumor cells. He et al. [83] constructed a novel FA-modified polymer carrier [Dioleoyl-3- trimethylammonium-propane (DOTAP)/FDMCA] with high transfection efficiency and safety. The carrier is composed of 1,2-DOTAP, FA-PEG-PCL, and MPEG-PLA, and can self-assemble with the MIP-3β plasmid to form a stable complex. The nanoparticles upregulated MIP-3β and promoted DC maturation, M1 polarization of macrophages, and activation of cytotoxic T cells in mouse models of subcutaneous and metastatic lung tumors, which significantly inhibited tumor growth and metastasis.

In the tumor microenvironment, CSF-1 can regulate the migration, proliferation, function, and survival of macrophages, and expands macrophage populations associated with cancer. The overexpression of CSF-1 and CSF-1R is often associated with poor tumor prognosis [84]. Therefore, CSF-1R blockade is an effective strategy against TAMs and several CSF-1R inhibitors are currently in the clinical testing phase [85]. Qian et al. [86] developed M2-type TAM-targeting nanoparticles (M2NPs) consisting of α-peptide (an SR-B1 targeting peptide) and M2 macrophage-targeting peptide [(M2pep), an M2 macrophage-binding peptide], and loaded a siRNA targeting CSF-1R on the M2NPs. The M2NPs effectively blocked the survival signal of M2-like TAMs and eliminated 52% of the TAMs in the B16F10 melanoma-xenograft mouse model, which significantly reduced tumor size (87%) and prolonged the survival of tumor-bearing mice. However, long-term inhibition of CSF-1R in tumors can reactivate macrophages through the IL-4 pathway, and drive resistance to CSF-1R through insulin-like growth factor-1 receptor (IGF-1R)/PI3K signaling, resulting in elevated STAT6 and nuclear factor of activated T cells (NFAT) [87]. On the other hand, combined inhibition of CSF-1R and PI3K showed a significant therapeutic effect on recurrent tumors. Therefore, gene therapy strategies targeting CSF-1R may need to be combined with other pathway inhibitors to achieve the best therapeutic effect.

IRFs are a class of transcription factors that are intracellular mediators of type I IFNs. They are important for hematopoietic development and immune processes [88]. Among them, IRF5 promotes M1 polarization of macrophages. Forced expression of IRF5 in M2 macrophages upregulates M1-specific cytokines, chemokines, and costimulatory molecules, and results in a potent Th1*/*Th17 response. Therefore, up-regulation of IRF5 expression in tumor macrophages may be an effective strategy for reprogramming the TAMs. For example, Zhang et al. [89] described a macrophages-targeting IRF5/IKKβ mRNA co-delivery system for the repolarization of TAMs. In order to improve the stability and translation ability of mRNA in the nanocarrier, the mRNA used was modified with ribonucleotides pseudouridine (Ψ) and 5-methylcytidine (m5C), which are capped with anti-reverse cap analog (ARCA). In addition, biodegradable cationic poly (β-amino ester) (PbAE) polymers were selected as carriers to form stable nano-complexes through electrostatic interactions with anionic mRNAs. Following the uptake of the nanoparticles by the target cells, the mRNA was released from the mRNA-PbAE complex by hydrolytic cleavage of ester bonds in the PbAE backbone. Finally, polyglutamic acid was used as a linker to attach a dimannitol fragment to the surface of the complex, which not only enabled the nano-complex to specifically target macrophages but also shielded the positive charges on the surface of PbAE-mRNA particles and enhanced their *in vivo* stability. Co-delivering the IRF5 and IKKβ mRNAs reprogrammed the immunosuppressive TAMs to an anti-tumor phenotype. This novel targeted delivery system can also be repeatedly and safely administered, and has high clinical potential.

## Conclusions

Macrophages are one of the most important components of the TME and establish complex tumor immunosuppressive microenvironment with stroma, cancer cells, and other immune cells, such as natural killer (NK) cells, B cells, and T cells. Macrophages could play a dual and opposing role in carcinogenesis and cancer progression. M1 macrophages participate in the regulation of T-cell proliferation, differentiation, and anti-tumor immunity formation. While M2 macrophages favor tumor growth, angiogenesis, and invasion, and suppress adaptive immunity. Therefore, the polarization of macrophages from the M2 to M1 phenotype can produce strong anti-tumor immunity, thus inhibiting tumor growth. Gene therapy, with the help of a delivery vehicle, represents a potential strategy to modulate macrophage polarization by activating or inhibiting cytokines and improving the efficiency of cancer immunotherapy. Current progress suggested the feasibility of targeting macrophages with nucleic acids for cancer treatment, this strategy still faces significant challenges for further clinical application and translation. Firstly, macrophage in the TME is a complex, diverse, and varied population with high heterogeneity, which is the major challenge to design carriers that can target specific macrophage populations and avoid being taken up by “unwanted” macrophages. Moreover, the phenotype of macrophages is highly dependent on the TME, which can easily reverse the phenotypic changes caused by the drug, thereby limiting the therapeutic efficacy. Also, delivery vectors can only transport certain nucleic acid therapeutics to macrophages at a particular site, however, the delivery efficiency of the same vectors is also heterogeneous in different cancer and different models. In addition, the complex interactions between macrophages and nanoparticles are still enigmatic in internalization, cytotoxicity, immune activation, and regulation, it is also critical to study nano/bio-interface events between nanomaterials and macrophages. Nonetheless, given the functional importance of macrophages and the advantages of nanoparticles, targeting macrophages have broad application prospects.

## Abbreviations

CSF-1: colony-stimulating factor-1
CSF-1R: colony-stimulating factor-1 receptor
FA: folic acid
IKKβ: inhibitor of kappa B kinase
IL-6: interleukin 6
IRF5: interferon regulatory factor 5
M2NPs: M2-type tumor-associated macrophage-targeting nanoparticles
Mincle: macrophage-inducible C-type lectin
MIP-3β: macrophage inflammatory response protein 3β
mRNA: messenger RNA
PbAE: poly (β-amino ester)
PD-L1: programmed cell death-ligand 1
pDNA: plasmid DNA
PI3K: phosphoinositide 3-kinase
shRNA: small hairpin RNA
siRNA: small interfering RNA
SMAD3: small mothers against decapentaplegic 3
STAT: signal transducer and activator of transcription
TAM: tumor-associated macrophage
TME: tumor microenvironment
TNF-α: tumor necrosis factor-α
TRMs: tissue-resident macrophages

## Declarations

### Copyright

© The Author(s) 2023.
